# Patients Offer Radiofrequency Ablation Therapy for Early Breast Cancer as Local Therapy (PO-RAFAELO) Study under the Patient-proposed Health Services

**DOI:** 10.31662/jmaj.2023-0047

**Published:** 2023-10-02

**Authors:** Shin Takayama, Takayuki Kinoshita, Sho Shiino, Kenjiro Jimbo, Ken-ichi Watanabe, Tomomi Fujisawa, Naohito Yamamoto, Tatsuya Onishi, Tadahiko Shien, Mitsuya Ito, Mina Takahashi, Manabu Futamura, Tomoyuki Aruga, Koji Kaneko, Akihiko Suto

**Affiliations:** 1 Department of Breast Surgery, National Cancer Center Hospital, Tokyo, Japan; 2 Department of Breast Surgery, National Hospital Organization Tokyo Medical Center, Tokyo, Japan; 3 Department of Breast Surgery, National Hospital Organization Hokkaido Cancer Center, Hokkaido, Japan; 4 Department of Breast Oncology, Gunma Prefectural Cancer Center, Gunma, Japan; 5 Division of Breast Surgery, Chiba Cancer Center, Chiba, Japan; 6 Department of Breast Surgery, National Cancer Center Hospital East, Chiba, Japan; 7 Department of Breast and Endocrine Surgery, Okayama University Hospital, Okayama, Japan; 8 Division of Breast Surgery, Hiroshima City Hiroshima Citizens Hospital, Hiroshima, Japan; 9 Department of Breast Oncology, National Hospital Organization Shikoku Cancer Center, Ehime, Japan; 10 Department of Breast Surgery, Gifu University Hospital, Gifu, Japan; 11 Department of Breast Surgery, Tokyo Metropolitan Cancer and Infectious Disease Center Komagome Hospital, Tokyo, Japan; 12 Department of Breast Oncology, Niigata Cancer Center Hospital, Niigata, Japan

**Keywords:** nonsurgical ablation, radiofrequency ablation therapy, breast cancer, Patient-proposed Health Services

## Abstract

**Introduction:**

Due to the increase in the number of early-stage breast cancer patients, there is growing interest in minimally invasive local therapies for breast cancer. Radiofrequency ablation (RFA) therapy is one of the most promising minimally invasive treatments. The Radiofrequency Ablation Therapy for Early Breast Cancer as Local Therapy (RAFAELO) study, a multicenter collaborative study that aims to validate the efficacy and safety of RFA and to standardize its use for early-stage breast cancer, was conducted under the Advanced Medical Care B system in 2013. This study enrolled the expected number of patients in November 2017; moreover, it is currently in the follow-up period. Some patients with early-stage breast cancer who are eligible for RFA could not receive the RFA treatment, as it is still not covered by insurance. Therefore, the Patients Offer Radiofrequency Ablation Therapy for Early Breast Cancer as Local Therapy (PO-RAFAELO) study under the Patient-proposed Health Services (PPHS) was proposed and approved in March 2019.

**Methods:**

The PPHS is a system that allows patients to receive prompt access to advanced medical care at a medical facility close to them, starting with their request. This system is considered a part of the specific and special medical coverage. The PO-RAFAELO study is the only study in the surgical field utilizing the PPHS, aiming to help in achieving regulatory approval and insurance coverage of RFA for breast cancer.

**Results:**

As of January 2023, 120 patients have undergone RFA using the PPHS and no grade 3 or higher early adverse events have occurred.

**Conclusions:**

A certain number of patients with early-stage breast cancer prefer nonsurgical treatment, and it is important to provide information regarding the availability of RFA for early-stage breast cancer under the PPHS.

Trial registration: registered with Japan Registry of Clinical Trial on March 06, 2019 (Trial ID: jRCTs032180187).

## Introduction

There is an increase in the number of patients with early-stage breast cancer due to recent improvements in diagnostic imaging. Therefore, minimally invasive local treatment has become a major research topic in breast cancer treatment. Minimally invasive treatments for breast cancer include radiofrequency ablation, cryoablation, and high-intensity focused ultrasound. Although such nonsurgical ablation has the potential to provide local control for limited indications, it should be implemented as clinical trials at this time and its implementation in actual clinical practice is premature. Radiofrequency ablation therapy (RFA) involves the insertion of an electrode needle into the lesion and the cauterization of the tumor site with a radiofrequency current, which is better as it causes minimal injury at the puncture site in the breast. Izzo et al. ^(1)^, Jeffery et al. ^(2)^, Burak et al. ^(3)^, and Kinoshita et al. ^(4), (5)^ demonstrated that RFA is effective and safe for breast cancer in a feasibility study of RFA followed by the resection of the thermal ablation site. Subsequently, Hayashi et al. ^(6)^, Fornage et al. ^(7)^, and Elliott et al.^(8)^ used only RFA for localized breast tumors with a diameter of ≤2 cm. Results showed that RFA could be a minimally invasive treatment option for breast cancer. Furthermore, Noguchi et al. ^(9)^, Esashi et al. ^(10)^, Yamamoto et al. ^(11)^, and Kinoshita et al. ^(4)^ reported the outcomes of RFA among patients with breast cancer in Japan, and some studies are still ongoing. However, there are no reports with sufficient statistical accuracy in Japan or other countries. Moreover, an appropriate pathological examination has not been established, and the eligibility criteria for RFA have not been developed.

A multicenter collaborative study was performed to validate the safety and efficacy of RFA and to standardize its use for early-stage breast cancer. The Radiofrequency Ablation Therapy for Early Breast Cancer as Local Therapy (RAFAELO) study was conducted under the Advanced Medical Care (AMC) B system in 2013. The RAFAELO study enrolled the expected number of patients (n = 372) in November 2017, and it is currently in the follow-up stage. In November 2021, the Japanese Breast Cancer Society requested the use of RFA for early-stage breast cancer, and this was considered suitable by the Ministry of Health, Labour and Welfare’s Study Group on the Early Introduction of Medical Devices with High Medical Needs. Prior to regulatory approval, the clinical positioning of RFA and the efficacy and safety of its medical devices must be validated based on the long-term efficacy and safety outcomes of the RAFAELO study. Thus, we are still working toward the regulatory approval and insurance coverage of RFA for early-stage breast cancer.

**Figure 1.**
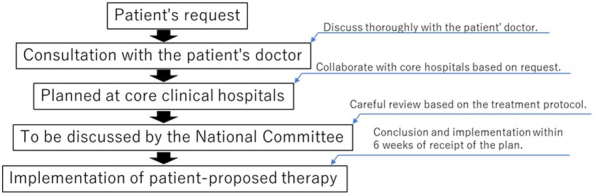
Process of the Patient-proposed Health Services The PPHS is designed to help patients receive advanced medical treatment promptly at medical institutions close to them, starting from their request.

However, patient enrollment in the RAFAELO study under the AMC B system was stopped in November 2017. Hence, some patients with early-stage breast cancer who are eligible for RFA cannot receive the treatment until it is covered by their insurance. Therefore, the Patients Offer Radiofrequency Ablation Therapy for Early Breast Cancer as Local Therapy (PO-RAFAELO) study, a multicenter collaborative research on the safety and efficacy of radiofrequency ablation therapy as a local therapy for early-stage breast cancer, under the Patient-proposed Health Services (PPHS) was proposed and approved in March 2019. The PPHS was launched in April 2016 and was proposed as a new mechanism within the mixed billing system to apply for a combination of treatment not covered by the public health insurance with treatment covered by the insurance ^(12)^. The difference between AMC and the PPHS is that AMC is planned by an investigator to examine the usefulness of the treatment whereas the PPHS is proposed by a patient to receive unapproved/off-label drugs ^(13)^. The PPHS allows the use of advanced medical treatment, which is not yet covered by social insurance but is intended to be in the future, combined with insured medical treatment under certain conditions, including the confirmation of treatment safety and efficacy, as a part of the special or specified medical care coverage. The system is designed to help patients receive such treatment promptly at medical institutions close to them upon their request ^(14)^. The PO-RAFAELO trial is a clinical trial initiated on request by a patient, and the clinical core hospital is required to prepare a treatment protocol, such as the preceding RAFAELO trial. Additionally, the safety and efficacy of the treatment after its completion must be confirmed by the government (Figure 1). It is challenging to achieve regulatory approval and insurance coverage based on this study alone. Nevertheless, if the preceding RAFAELO study confirms the efficacy and safety of RFA and if the PO-RAFAELO study shows that RFA can be safely performed at more facilities, the results can be used as a basis for the regulatory approval and insurance coverage of RFA. As of January 2023, seven treatments have been approved under the PPHS but the PO-RAFAELO trial is the only study approved in the surgical area (Table 1).

**Table 1.** Overview of Each Technology for the Patient-Proposed Health Services.

Registration No.	Medical technology of patient-offered therapy	Diseases for which a specific medicine is indicated
1.	Combination therapy of intraperitoneal and intravenous paclitaxel and oral S-1	Peritoneal dissemination or advanced gastric cancer
2.	-	-
3.	Oral infigratinib therapy	Advanced solid tumors
4.	Percutaneous radiofrequency ablation of breast cancer	Early-stage breast cancer (limited to breast cancer with a long diameter of 1.5 cm or less)
5.	Molecular targeted therapy based on gene profiling by multiplex gene panel test	Advanced solid tumors that cannot be curatively resected
6.	Intravenous trastuzumab emtansine	Extramammary Paget’s disease
7.	-	-
8.	Intravenous rituximab	Refractory chronic inflammatory demyelinating polyneuropathy
9.	Combination therapy with oral dabrafenib and oral trametinib	Glioma

## Regarding the RAFAELO Study

The primary endpoint of the RAFAELO study is 5-year disease-free survival of the ipsilateral breast, and the secondary endpoints are incomplete ablation rate, overall survival, distant recurrence-free survival, and adverse events. Follow-up, including medical examinations and imaging studies, is performed at 6-month intervals from 1 to 5 years after RFA. Furthermore, the follow-up includes evaluation of esthetic outcomes by photographs and by questionnaire. Unlike the PO-RAFAELO study, the RAFAELO study includes an assessment of long-term prognosis and esthetic assessment. There are no major differences between the two studies with respect to eligibility criteria, treatment protocol, and criteria for treatment completion ^(15)^.

## Goal of the PO-RAFAELO Study

This study aimed to gather data on safety in the multifacility study treatment (RFA) in Japan. Further, we collected information on the efficacy of the treatment provided in this study. These endpoints were established to provide useful information that could complement the results of the RAFAELO trial for the future dissemination of the treatment of this study (RFA). Patients who met the eligibility criteria could undergo total mastectomy or breast-conserving surgery covered by insurance but could refuse if they do not want to undergo surgical resection or remain cosmetically intact. This RFA treatment in the PPHS is no evidence of its safety or efficacy currently. Additionally, it is not a standard treatment, although it has been provided as an advanced medical treatment. However, the implementation of this RFA treatment is deemed appropriate for the following reasons.

(1) Patient enrollment for the RAFAELO study of Advanced Medical Treatment B, which was conducted prior to the study, was terminated. Hence, patients are no longer able to participate in the advanced medical treatment for RFA.

(2) Although the main analysis of the preceding RAFAELO trial has not been conducted, RFA itself is not an invalid treatment for cancer. Therefore, there is little concern that it will cause a notable disadvantage to patients even if it is implemented as the PPHS under careful observation.

(3) In the RAFAELO study, no serious adverse events were observed in the short term.

Obtaining regulatory approval and insurance coverage based on this study alone is difficult. However, if the effectiveness and safety of RFA are demonstrated in the preceding RAFAELO study and if this study confirms the safety of RFA performed at multiple centers, the results of the PO-RAFAELO study will be used in combination with those of the RAFAELO study to obtain regulatory approval and insurance coverage.

## Endpoint

Primary endpoint: incidence rate of ≥grade 3 early adverse events.

The denominator is the total number of patients treated, and the numerator is the number of patients who had at least one nonhematologic toxicity (≥grade 3). An adverse event is defined as the presence of ≥grade 3 adverse events according to the Common Terminology Criteria for Adverse Events version 4.0 or the Japan Clinical Oncology Group postoperative complication criteria (Clavien-Dindo classification). Early adverse events may be a potential indicator of RFA safety.

Secondary endpoints: rates of incomplete ablation and treatment completion.

Incomplete ablation is defined as the presence of residual viable tumor cells on vacuum-assisted biopsy. Additional surgical excision for incomplete ablation may be expected to provide local control comparable to standard surgery. Thus, the percentage of incomplete ablation may be an indicator of the efficacy of RFA.

Treatment completion is defined as treatment completion based on the study protocol, which used the total number of patients treated in the trial as the denominator. Thus, treatment completion is an indicator of whether the treatment was performed according to the protocol and is important in determining the quality of the trial.

## Study Design

**Figure 2.**
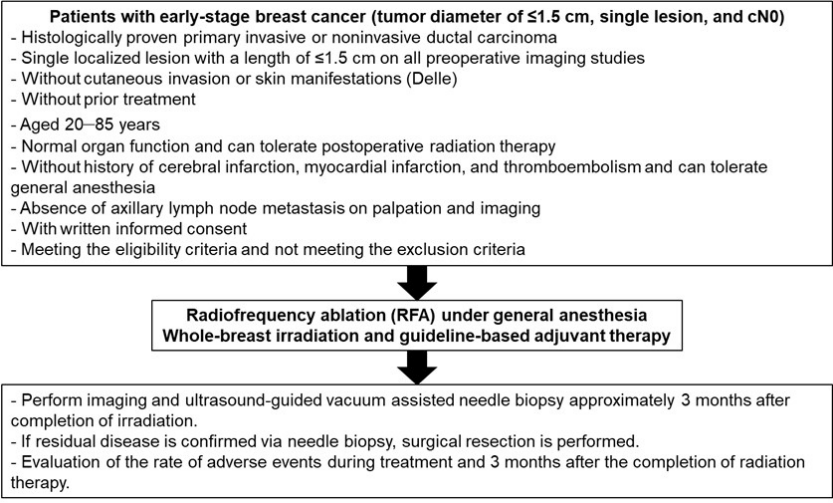
Schema of patient inclusion and treatment in the PO-RAFAELO study.

This uncontrolled study aimed to evaluate the safety and efficacy of RFA in patients with early-stage breast cancer (tumor diameter: ≤1.5 cm) (Figure 2).

## Eligibility Criteria

### Inclusion criteria

1) Patients with primary invasive or noninvasive ductal carcinoma of the breast histologically confirmed via needle biopsy.

2) Patients with a single localized lesion with a diameter of ≤1.5 cm on all preoperative imaging studies.

3) Patients without skin invasion or other skin findings.

4) Patients without prior treatment (such as chemotherapy, hormone therapy, and radiotherapy) for the current lesion.

5) Women aged 20-85 years.

6）Patients who have normal organ function and who can tolerate postoperative radiation therapy.

7) Patients who do not have a previous history of cerebral infarction, myocardial infarction, thromboembolism, and those who can tolerate general anesthesia.

8) Patients without axillary lymph node metastasis evident on palpation and diagnostic imaging.

9) Patients who provided written informed consent.

10) Patients who meet the eligibility criteria and none of the exclusion criteria during enrollment.

### Exclusion criteria

1) Patients who are pregnant or possibly pregnant.

2) Patients with a cardiac pacemaker or an implantable cardioverter-defibrillator.

3) Patients with complicated local active inflammation or infection.

4) Patients with major cardiac or cerebral disease.

5) Patients with artificial bone or other implants that can prevent the application of a counter plate and that are contraindicated to RFA.

6) Patients receiving treatment, such as antiplatelet and anticoagulation therapy, that can affect hemostasis.

7) Patients with extensive intraductal breast lesions or suspected multiple lesions on imaging.

8) Patients with extensive calcification on mammogram.

9) Patients with ectopic ipsilateral breast cancer with recurrence in the preserved breast.

10) Patients with other organ metastases.

11) Patients who are not eligible for this study based on the discretion of the attending physician.

## Treatment Protocol

RFA is a technique in which a radiofrequency electrode needle is inserted into an intramammary lesion from the breast surface through breast ultrasonography guidance and radiofrequency thermal ablation is performed on the lesion.

### Generic name of the medical device: Radiofrequency ablation system

Brand name of the medical device: Cool-tip RF System or Cool-tip RFA System E Series

RFA: Under general anesthesia, an ultrasound-guided radiofrequency electrode needle is inserted into an intramammary lesion from the breast surface, and the lesion is cauterized with radiofrequency heat.

### Treatment of the axilla

Sentinel lymph node biopsy is recommended prior to RFA. An intraoperative pathological assessment is performed to confirm the presence or absence of metastases, but RFA should be performed as scheduled regardless of the presence or absence of metastases. If axillary node metastasis is positive, the decision to perform axillary lymph node dissection should be made as in the case of partial mastectomy.

Radiotherapy: All patients required 40-50 Gy at 1.8-2 Gy per fraction (total: 25 doses) within 8 weeks after RFA completion or within 8 weeks after adjuvant chemotherapy completion. Additional boost irradiation of up to 10 Gy at the center of the RFA treatment site may be provided based on the discretion of the attending physician.

Surgical resection: If the tumor is viable based on a pathological study due to needle biopsy performed 3 months after the completion of radiation, partial or total mastectomy should be performed.

Postoperative adjuvant therapy: Postoperative adjuvant therapy should be administered according to the standard of care. In hormone receptor-positive patients, endocrine therapy should be initiated after or concurrent with radiation therapy and administered within an appropriate duration. Moreover, postoperative chemotherapy regimens should be determined according to the standard of care and administered before radiation if necessary. Postoperative adjuvant therapy is not included in the treatment protocol.

## Criteria for Treatment Completion

Treatment is considered complete if RFA and subsequent radiation therapy are performed as prescribed. Next, imaging and needle biopsy are conducted within 3 months after radiation therapy completion to confirm the absence of residual disease. Even if needle biopsy shows residual disease, treatment is considered complete if additional surgery (partial or total mastectomy) is performed immediately. The date of treatment completion is defined as the date when the absence of a residual lesion is confirmed via needle biopsy or the date when additional surgery is performed.

## Follow-up and Laboratory Tests before Registration and during Follow-up after RFA

Before study registration, palpation, mammography, breast ultrasonography, and breast MRI will be performed to confirm the tumor diameter (<1.5 cm) in all cases. After RFA is performed, the presence or absence of abnormalities and adverse events at the site of RFA in the breast should be confirmed via interview and palpation. Adverse events will be evaluated using the Common Terminology Criteria for Adverse Events version 4.0 or the Japan Clinical Oncology Group postoperative complication criteria (Clavien-Dindo classification). Approximately 3 months after the completion of radiation, breast ultrasonography, breast MRI, and vacuum-assisted biopsy should be performed to determine if there are any residual tumor cells (Table 2).

**Table 2.** Schedule of Follow-up after RFA in the PO-RAFAELO Study

	Preregistration	Intraoperative/ postoperative	Within 1 week after RFA	Approximately 3 months after radiation completion
Patient characteristics: current and previous medical history	〇			
Interview	〇		〇	〇
Visual assessment and palpation	〇	〇		〇
MG	〇			
CNB* or VAB	〇			〇
Breast US	〇	〇		〇
Breast MRI and/or CT scan	〇			〇
Chest radiography	〇			
Tumor marker evaluation	〇			〇

* Core needle biopsy (CNB) is acceptable only for preoperative diagnosis.

## Statistical Analysis

### Rationale for setting the study period and enrollment

The planned study duration is 6 years. As the study is based on patient requests, the number of enrollees will not be set based on statistical grounds and analysis will be conducted on patients who were enrolled during the 6-year enrollment period. In the preceding clinical trial (RAFAELO study) under the AMC B system, 372 patients were enrolled over a 5-year period or approximately 70 patients were included annually. Based on this result, approximately 50 patients will be enrolled annually. If the enrollment period is 6 years and a total of 300 patients are enrolled, the 95% confidence interval of ≥grade 3 early adverse events will be 0.2%-2.9% if the rate is 1% (3/300 patients).

Enrollment period: 6 years

Follow-up period: 4 months after radiotherapy

Start of the study: March 1, 2019

Period: from March 1, 2019, to February 28, 2026

Analysis period: 1 year after the end of the follow-up period

Study duration: 8 years from the date of study approval

## Clinical Hypothesis

Although RFA is expected to be less invasive and more cosmetic than surgical treatment ^(16)^, data on its safety and efficacy are currently limited. Therefore, this study hypothesized that RFA is not inferior to the standard treatment in terms of early adverse events. If the RAFAELO study shows that RFA is effective and safe and if the data from both RAFAELO and PO-RAFAELO studies support the abovementioned hypothesis, then RFA will be considered for early-stage breast cancer (tumors with a diameter of ≤1.5 cm).

The most common early adverse event associated with partial mastectomy, the standard treatment for early-stage breast cancer, is infection, with incidence rates of 3.8% ^(17)^ and 1.97% ^(18)^ based on previous studies. The former report was made at an earlier date, and the incidence of infections or complications is predicted to have declined in recent years. The latter report is based on the complication rate at 30 days postoperatively, and if the observation period is extended to 3 months, the complication rate is considered somewhat higher. Considering this, the early adverse events of standard treatment will be 3%, and the incidence of early adverse events in this study will not be significantly higher than that value.

## Interim Analysis and Discontinuation of the Study

As the primary endpoint of this study is the incidence rate of ≥grade 3 adverse events and treatment safety, an interim analysis will not be performed. The criteria for study discontinuation based on safety endpoint were defined separately. In the event that the safety endpoint is exceeded, this will be reported to the Efficacy and Safety Evaluation Committee and the study will be discontinued. The results will also be reported to the Patient-designated Therapy Evaluation Committee, which will decide whether to continue the treatment as a patient-designated therapy.

## Final Analysis

After the follow-up period, all endpoints will be analyzed after the data are confirmed based on the final survey. The incidence rate of ≥grade 3 early adverse events and the rates of incomplete ablation and treatment completion will be calculated. Next, the exact 95% confidence interval based on the binomial distribution will be calculated. This study will not perform statistical analyses for validation purposes.

The incidence rate of ≥grade 3 early adverse events, which is the primary endpoint, will not exceed 5%. The secondary endpoint was also set for obtaining information that will be helpful for the future dissemination of study treatment in this trial. The expected rates of incomplete ablation and treatment completion are approximately 15% and 90%, respectively.

## Significance of the Study

If nonsurgical resection such as RFA can provide local control of early-stage breast cancer with a tumor diameter of 1.5 cm or less, it may provide an alternative to partial or total mastectomy. Currently, RFA therapy for breast cancer is not covered by insurance, but it can be performed by participating in the PO-RAFAELO trial under the PPHS. As of January 2023, 120 patients have undergone RFA using the PPHS and no grade 3 or higher early adverse events have occurred. The PO-RAFAELO study is the only study in the surgical field utilizing the PPHS, aiming to help in achieving regulatory approval and insurance coverage of RFA for breast cancer. It is important to provide information regarding the availability of RFA for early-stage breast cancer under the PPHS.

## Article Information

### Conflicts of Interest

None

### Sources of Funding

This work was supported by, in accordance with the Patient-proposed Health Services, the medical fees paid by patients registered in the study to the implementing medical institution, which were then remitted to the National Cancer Center by the implementing medical institution in accordance with the contract for operating expenses.

### Acknowledgement

The authors would like to thank Enago (www.enago.jp) for English language review.

### Author Contributions

Study conception: ST and TK. Study design: ST, TK, SS, and KJ. Study conduct: ST, TK, SS, KJ, KW, TF, TO, TS, MF, and TA. Drafting of the manuscript: ST, TK, and SS. Critical revision of the manuscript for important intellectual content: ST, TK, TF, NY, MI, MT, KK, and AS. The authors read and approved the final manuscript.

### Approval by Institutional Review Board (IRB)

The clinical trial received institutional review board (IRB) approval from the IRB committees of National Cancer Center Hospital (CRB3180008). It was registered with Japan Registry of Clinical Trial on March 06, 2019 (Trial ID: jRCTs032180187). Written informed consent to participate in the trial was obtained from all participants.

### The Following Institutions Participated in the Study

National Cancer Center Hospital, National Hospital Organization Tokyo Medical Center, National Hospital Organization Hokkaido Cancer Center, Gunma Prefectural Cancer Center, Chiba Cancer Center, National Cancer Center Hospital East, Okayama University Hospital, Hiroshima City Hiroshima Citizens Hospital, National Hospital Organization Shikoku Cancer Center, Gifu University Hospital, Tokyo Metropolitan Cancer and Infectious Disease Center Komagome Hospital, and Niigata Cancer Center Hospital
